# First Detection of *Wolbachia* in Namibian Bird Ectoparasites (Acariformes: Syringophilidae) with a Description of New Quill Mite Species †

**DOI:** 10.3390/ani15010052

**Published:** 2024-12-28

**Authors:** Eliza Glowska-Patyniak, Katarzyna Kaszewska-Gilas, Izabella Laniecka, Julia Olechnowicz, Kamila Ostrowska, Wiktoria Dmuchowska, Brian K. Schmidt, Jan Hubert, Artur Trzebny

**Affiliations:** 1 Department of Animal Morphology, Faculty of Biology, Adam Mickiewicz University in Poznań, Uniwersytetu Poznańskiego 6, 61-614 Poznań, Poland; k.kaszewska@amu.edu.pl (K.K.-G.); izabella.laniecka@gmail.com (I.L.); kamromanowska@gmail.com (K.O.); wikdmu@st.amu.edu.pl (W.D.); 2 Molecular Biology Techniques Laboratory, Faculty of Biology, Adam Mickiewicz University in Poznań, Uniwersytetu Poznańskiego 6, 61-614 Poznań, Poland; julia.olechnowicz@amu.edu.pl (J.O.); arturtrzebny@amu.edu.pl (A.T.); 3 Division of Birds, Smithsonian Institution, P.O. Box 37012, MRC 116, Washington, DC 20013-7012, USA; schmidtb@si.edu; 4 Crop Research Institute, Drnovska 507/73, 6-Ruzyne, CZ-16106 Prague, Czech Republic; carpoglyphus@gmail.com

**Keywords:** *Wolbachia*, quill mites, bird parasites, molecular taxonomy, 16S rRNA gene amplicon sequencing, COI

## Abstract

*Wolbachia* is a common intracellular bacterial genus that infects numerous arthropods and filarial nematodes. In arthropods, it typically acts as a reproductive parasite, leading to various phenotypic effects such as cytoplasmic incompatibility, parthenogenesis, feminization, or male-killing. Quill mites (Acariformes: Syringophilidae) are a group of bird parasites that have recently attracted increasing interest due to the detection of unique phylogenetic lineages of endosymbiotic bacteria and potentially pathogenic taxa. Our study used an unbiased 16S rRNA gene amplicon sequencing approach to examine several populations of Namibian quill mites for the presence of bacteria that could affect their biology. We detected *Wolbachia* in two mite populations collected from two species of larks. However, we did not find any other endosymbiotic bacteria or any that could be of epidemiological importance. Since the mite taxa we tested were previously unknown to science, we conducted comprehensive morphological and molecular systematic analyses on them. Our research revealed two new quill mite species of the genus *Syringophilopsis* Kethley, 1970 which parasitize three sub-Saharan alaudids, i.e., *Syringophilopsis erythrochlamys* sp. n. from the dune lark *Calendulauda erythrochlamys* (Strickland, HE) and *S. christinae* sp. n. from the Karoo long-billed lark *Certhilauda subcoronata* (Smith) and spike-heeled lark *Chersomanes albofasciata* (de Lafresnaye, NFAA). In addition, we provided the African reed warbler *Acrocephalus baeticatus* (Vieillot, LJP) as a new host for *S. acrocephali* Skoracki, 1999. Our study expands the knowledge on parasite diversity and provides new insights into *Wolbachia* infection among quill mites in Africa.

## Simple Summary

We report the first detection of *Wolbachia* in the quill mites parasitizing African birds. The further findings of our study are two new-to-science syringophilid species living on alaudid birds in Namibia, i.e., *Syringophilopsis erythrochlamys* sp. n. from the dune lark *Calendulauda erythrochlamys,* (Strickland, HE) and *Syringophilopsis christinae* sp. n. from the Karoo long-billed lark *Certhilauda subcoronata* (Smith) and the spike-heeled lark *Chersomanes albofasciata* (de Lafresnaye, NFAA). In addition, we provide the African reed warbler *Acrocephalus baeticatus* (Vieillot, LJP) as a new host for *S. acrocephali* (Skoracki, 1999). Our discovery broadens the understanding of parasite diversity and offers the latest insights into *Wolbachia* infection among quill mites.

## 1. Introduction

*Wolbachia* is the most abundant intracellular bacterial genus, infecting a wide range of arthropods and filarial nematodes. In arthropods, bacteria typically function as reproductive parasites, leading to various phenotypic effects such as cytoplasmic incompatibility, parthenogenesis, feminization, or male-killing [1,2,3]. One of the groups of organisms in which a large diversity of strains of this endosymbiont has been detected is quill mites (Acariformes: Syringophilidae) [4,5].

Syringophilids are widespread but still poorly known bird ectoparasites. These organisms’ entire life cycle occurs within the bird’s feather (inside the quill). The diversity of this family is estimated to be 5000 species [6], but no more than 10% of these have been discovered and described so far [7,8]. Despite the gradual increase in knowledge about their species richness and host associations [9,10], they remain among the least-known bird parasites in terms of their biology, impact on the avian host, and potential epidemiological significance. These difficulties have resulted from the nature of mites themselves (small body size, hard-to-reach habitat, relatively low prevalence, and weakly informative morphology) [11,12], but also from the limited research methodologies that can be applied. Much of the research has been conducted on museum collections, but material stored in this way is largely unsuitable for molecular analyses, thus limiting research methods to morphological tools.

In recent years, combined descriptions have been increasingly proposed in the systematics of quill mites. They are based on classical morphological data and complemented by DNA barcoding (short fragment of the mitochondrial cytochrome c oxidase subunit I sequence, COI). Although only a small fraction of quill mite diversity has been barcoded so far, it has revealed phenomena such as phenotypic plasticity [12], female dimorphism [13], and cryptic species [14]. Thus, it has proved its usefulness in this type of research. Precise and unambiguous species diagnosis, extended by quill mites’ parasitological and epidemiological significance, is essential for all research. This is particularly important in the context of recent reports indicating that mites host very diverse and largely unique phylogenetic lineages of endosymbiotic bacteria from the genera *Wolbachia* and *Spiroplasma*, which may be responsible for the strong unequal sex ratio (over-representation of females) observed in most mite species [4,5]. Due to the way they take in food (piercing the quill wall and sucking the bird’s body fluids), mites are considered a potential vector of infectious diseases between birds and have been found to harbour *Anaplasma*, *Bartonella,* and *Brucella* taxa [5,15]. Our understanding of the relationships between quill mites and their endosymbionts or pathogens has primarily been derived from studies of mites that parasitize passerine birds in Poland. Considering the significant diversity observed among several mite species in a single location, expanding research to include other bird taxa from different zoogeographic regions could significantly enhance our knowledge of the distribution and diversity of these bacteria.

To address the above, we used an unbiased 16S rRNA gene amplicon sequencing method to investigate four populations of Namibian quill mites originating from four bird species for the presence of bacteria that could impact their biology and have epidemiological significance. Since the mite taxa we tested were previously unknown to science, we also conducted morphological and molecular systematic analyses.

## 2. Materials and Methods

### 2.1. Animal Material

The mite material used in the study (Table 1) was acquired from the feather collection deposited in the Smithsonian Institution, National Museum of Natural History, Department of Vertebrate Zoology, Division of Birds, Washington, DC, USA (USNM). The birds were originally collected in Namibia, Karas Province, in August 2009 (coll. Gebhard, C. A., Schmidt, B. K., and Komen, J.). The feathers in the collection were separated from the birds’ bodies, dried, and stored in envelopes at room temperature. Mite material was obtained in September 2013 (coll. Eliza Głowska-Patyniak) from the feathers of four different bird species, i.e., the Karoo long-billed lark, spike-heeled lark, dune lark, and African reedwarbler. Mite-infected quills/feathers were placed in 96% ethanol and frozen. One secondary flight feather was analyzed from each bird specimen and dissected under a stereo microscope (Olympus ZS30). Individual mites were washed twice, preserved in 96% ethanol, and forwarded for non-invasive DNA isolation. This procedure left the exoskeletons intact and, after DNA extraction, the specimens were mounted on microscopic slides in a Faure medium.

### 2.2. Molecular Data and Analysis

#### 2.2.1. DNA Extraction

Total genomic DNA was extracted from single specimens using the DNeasy Blood & Tissue Kit (Qiagen GmbH, Hilden, Germany), as described by Dabert et al. [16]. To identify potential contaminants, in addition to sequencing a negative control alongside all samples, we further extracted DNA from the reagents and materials commonly used in the laboratory in which this work was carried out. Each library was created from an extraction buffer (ALT), millipore water, microscope swabs, pipette swabs, and other equipment (pincettes, scalpels, benches, etc.). These five libraries were processed and sequenced separately from the other samples using identical procedures. All DNA samples and corresponding voucher specimens have been deposited in the collection of the Department of Animal Morphology, Faculty of Biology, Adam Mickiewicz University in Poznan, Poland.

#### 2.2.2. Library Preparation and Sequencing

The V4 hypervariable region of the 16S rRNA gene was amplified using PCR primers V4F (GATCAGCAGCCGCGGTAATA) and V4R (GGACTACCAGGGTATCTAA) [17], fused with indexes and ion torrent adapters as in [5]. For the PCRs, each 10 μL sample was prepared in two technical replicates containing 1× HOT FIREPol Blend Master Mix (Solis BioDyne), 0.25 μM of each double-indexed fusion primer, and about 1 ng of template DNA. The fusion PCR regime used was 12 min at 95 °C, 30 cycles of 15 s at 95 °C, 90 s at 50 °C, 30 s at 72 °C, and a final 5 min at 72 °C. After the PCR, all samples were pooled, size-selected on a E-gel™ SizeSelect™ 2% Agarose Gel (Invitrogen by Thermofisher Scientific, Waltham, MA, USA), according to the manufacturer’s protocols. The DNA concentrations and the length of the amplicons were quantified on a 2200 TapeStation (Agilent Technologies, Inc., Santa Clara, CA, USA). Clonal template amplification on the ion sphere particles (ISPs) was performed using the Ion Torrent One Touch System II and the Ion 540™ Kit-OT2 with regard to the manufacturer’s instructions. Sequencing of the templated ISPs was conducted on the Ion 540™ Chip with the use of Ion 540™ sequencing chemistry and the Ion Gene Studio S5™ System (Ion Torrent, Thermo Fisher Scientific, Inc., Waltham, MA, USA) at the Molecular Biology Techniques Laboratory, Faculty of Biology, AMU. The data that support the findings of this study are openly available from NCBI under the BioProject accession number PRJNA1193096.

#### 2.2.3. Read Processing and Statistical Analyses

Raw sequencing data were prefiltered by Ion Torrent Suite software version 5.18.1 (Life Technologies, Carlsbad, CA, USA) to remove polyclonal and low-quality sequences. Further bioinformatic analysis was conducted using fastq data and a custom workflow. Sequence reads shorter than 180 bp were removed from the dataset using Geneious Prime 2023.1.2 (Biomatters Ltd., Auckland, New Zealand). The FastX-Toolkit [18] was used to extract sequences, with a minimum of 50% of bases having a quality score ≥ 25. Quality filtered sequences were separated into individual combinations of indexes in Geneious Prime. Next, the sequences were trimmed at the 5′ and 3′ ends to exclude the PCR primers. Then, sequences were denoised to generate amplicon sequencing variants (ASVs) using the DADA2 denoise-pyro method implemented in QIIME2 version 2024.2 [19,20]. The UNCROSS2 algorithm was used to remove ASVs detected in the control samples from the dataset [21]. Then, ASVs were compared against the SILVA database for ARB for small subunit ribosomal RNAs version 138.1 [www.arb-silva.de] [22,23,24].

#### 2.2.4. COI Sequences

The COI gene fragment was amplified by PCR with the degenerate primers Aseq01F (GGAACRATATAYTTTATTTTTAGA) and Aseq03R (GGATCTCCWCCTCCWGATGGATT) [13]. PCR amplifications were carried out in 10 µL reaction volumes containing 5 µL of Type-it Microsatellite Kit (Qiagen), 0.5 µM of each primer, and 4 µL of the DNA template using a thermocycling profile of one cycle of 5 min at 95 °C, followed by 35 steps of 30 s at 95 °C, 1 min at 50 °C, 1 min at 72 °C, with a final step of 5 min at 72 °C. After amplification, the PCR products were diluted two-fold with MQ water, and 5 µL of the sample was analyzed by electrophoresis on a 1.0% agarose gel. Samples containing visible bands were purified with thermosensitive Exonuclease I and FastAP alkaline phosphatase (Fermentas, Thermo Scientific). The amplicons (565 bp) were sequenced in one direction using the Aseq01F primer. Sequencing was performed with BigDye Terminator v3.1 on an ABI Prism 3130XL Analyzer (Applied Biosystems, Foster City, CA, USA). Sequence chromatograms were checked for accuracy and edited using Geneious R11 (Biomatters Ltd.). Pairwise distances between the nucleotide COI sequences were calculated using Kimura’s two-parameter (K2P) and distance p models for all codon positions with MEGA7 [25,26].

### 2.3. Morphological Analysis

All morphological observations and species identification were performed with an Olympus BH2 microscope with differential interference contrast (DIC) optics and a camera lucida. All measurements are in micrometers (µm). The idiosomal setation follows [27], with modifications adapted for the Prostigmata by [28]. The nomenclature of leg chaetotaxy follows that proposed by [29]. The application of this chaetotaxy to Syringophilidae was provided by Bochkov et al. [30], with a few changes made by [31]. The latin and common names of the birds follow those by Clements et al. [32].

Material depositories and abbreviations: AMU—Adam Mickiewicz University in Poznań, Poznań, Poland; USNM—Smithsonian Institution, National Museum of Natural History, Washington, DC, USA. The voucher slides and corresponding DNA samples are deposited in the AMU collection using the identification numbers indicated below. The sequences are deposited in GenBank under the accession nos. specified below.

## 3. Results

### 3.1. Microbial Composition

**Figure 1:**
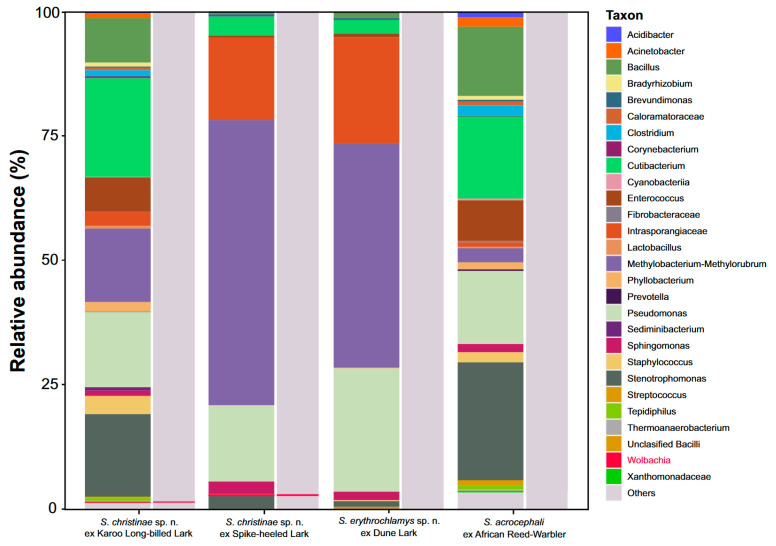
Overview of the bacterial taxa detected in quill mites. Bar plots show the most abundant bacterial taxa. Each stacked bar represents a bacterial community obtained from the mites collected from one bird species. The height of stacks represents the relative abundances of each taxon.

**Figure 2:**
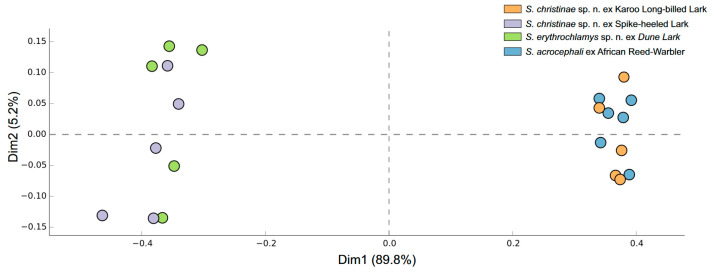
Principal coordinates analysis (PCoA) based on Bray–Curtis distance showing the microbial composition of quill mites. Each point represents a sample and the spatial arrangement reflects the similarity in microbial community structure.

We investigated the microbial composition of 21 individuals of three quill mite species originating from four bird host species (Table 1). After processing the reads, 153 ASVs were denoised. Among the most abundant genera, 98 ASVs were clustered (Figure 1 and Figure 2). Bar plots of ASV abundance and ordination analyses revealed similar microbiome compositions, but the relative abundance of particular bacterial taxa differed across the mite species/populations and formed two distinct groups. The first one comprises microbial associates found in *S. christinae* sp. n. population from the Karoo long-billed lark and *S. acrocephali* from the African reed warbler. In these populations, we observed the highest proportion of bacteria from the following genera: *Cutibacterium*, *Stenotrophomonas*, *Pseudomonas*, *Bacillus*, *Enterococcus,* and *Methylobacterium*. In the population of *S. christinae* sp. n. from the spike-heeled Lark and *S. erythrochlamys* sp. n. from the dune lark, the highest relative abundance was recorded for *Methylobacterium*, Intrasporangiaceae, and *Pseudomonas*.

We detected *Wolbachia* in four individuals of *S. christinae* sp. n., collected from both species of larks (Karoo long-billed lark and spike-heeled lark) with a relative abundance of 0.5% (Figure 1). We did not find any other ASVs that could be assigned to other previously known endosymbiotic bacteria or bacterial taxa of epidemiological importance.

### 3.2. Systematics

Family: Syringophilidae Lavoipierre
Subfamily: Syringophilinae Lavoipierre
Genus *Syringophilopsis* Kethley

#### 3.2.1. Molecular Data

We analyzed all available materials for the molecular studies, but we only obtained high-quality COI sequences for two out of the three species., i.e., *Syringophilopsis erythrochlamys* sp. n. and *S. acrocephali*. The COI alignment was 565 bp long and comprised six sequences (two of *S. erythrochlamys* sp. n. and four of *S. acrocephali*), with one haplotype representation for each species. The alignment contained 77 variable sites. The intraspecific genetic distances were 0.0%, and the interspecific values were 13.6% and 15.1% for distance *p* and the Kimura two-parameter, respectively.

#### 3.2.2. Morphological Systematics

##### Descriptions

**Figure 3:**
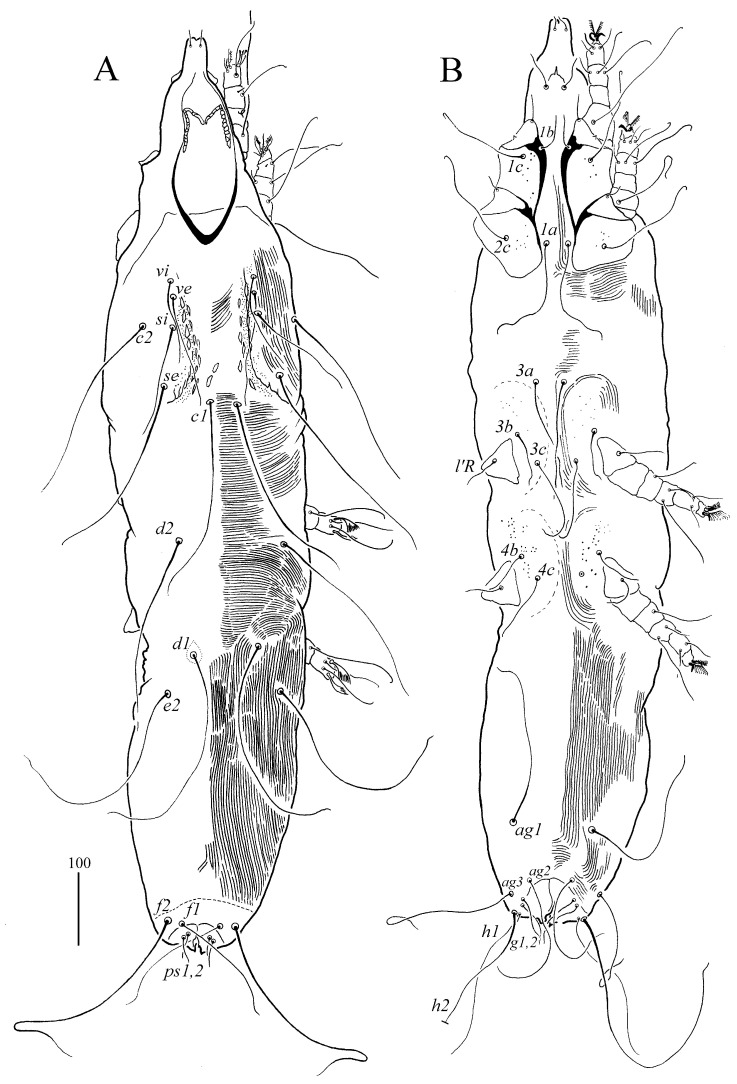
(**A**,**B**) *Syringophilopsis erythrochlamys* sp. n., female: (**A**) dorsal view, (**B**) ventral view.

**Figure 4:**
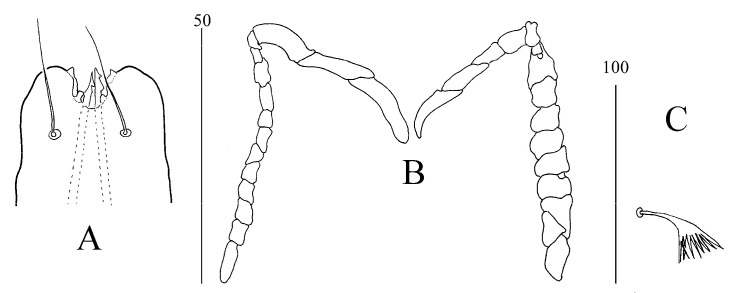
(**A**–**C**) *Syringophilopsis erythrochlamys* sp. n., female: (**A**) hypostomal apex (50 µm), (**B**) peritremes (50 µm), (**C**) fan-like setae p′ of leg III (100 µm).

**Figure 5:**
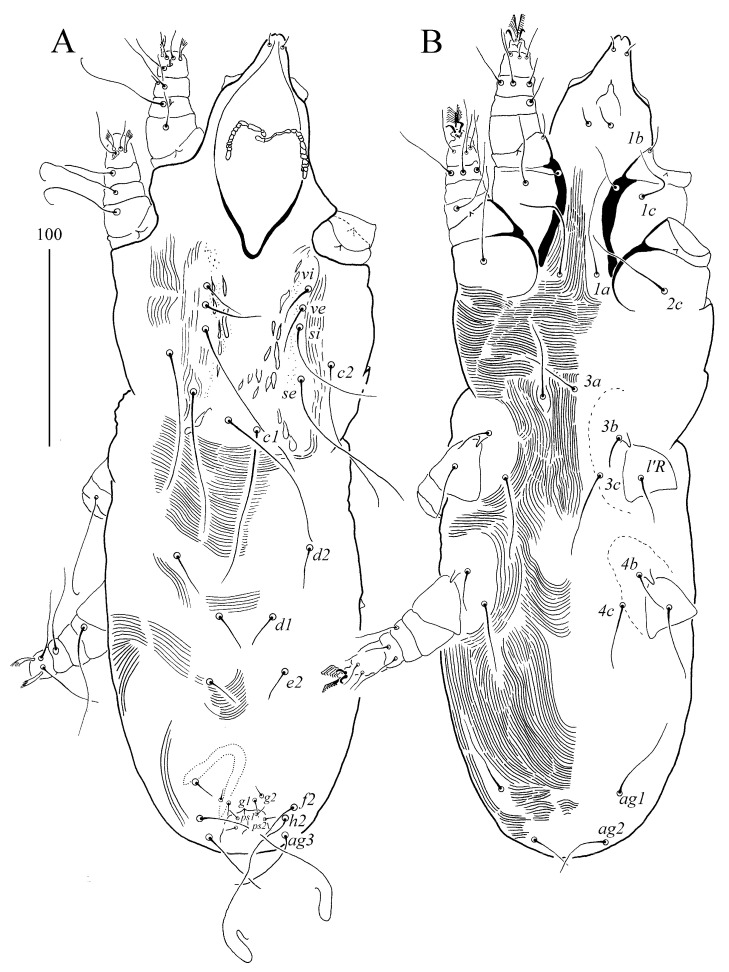
(**A**,**B**) *Syringophilopsis erythrochlamys* sp. n., male: (**A**) dorsal view, (**B**) ventral view.

**Figure 6:**
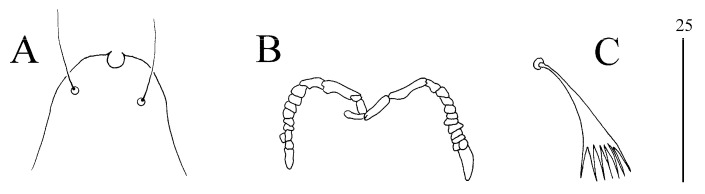
(**A**–**C**) *Syringophilopsis erythrochlamys* sp. n., male: (**A**) hypostomal apex, (**B**) peritremes, (**C**) fan-like setae p′ of leg III.

*Syringophilopsis erythrochlamys* sp. n. (Figure 3, Figure 4, Figure 5 and Figure 6)

Female (holotype and 10 paratypes, range in parentheses) (Figure 3 and Figure 4). Total body length: 1320 (1185–1285 in 10 paratypes). *Gnathosoma*. Hypostomal apex with protuberances. Infracapitulum apunctate. Each medial branch has 3–4 chambers, each lateral branch has 10–12 chambers. Length of stylophore and movable cheliceral digit: 255 (240–245) and 220 (170–230), respectively. *Idiosoma.* Propodonotal shield with deeply concave anterior margin, punctate and visibly sculptured on the lateral and central part of shield, bearing bases of setae *vi*, *ve*, and *si*. Bases of setae *c1* are situated posterior to level *se*, slightly out of the propodonotal shield. The bases of setae *si* and *c2* are situated at the same transverse level. Length ratio of setae *vi*:*ve*:*si* 1:1.3–1.4:2.2–2.5. The hysteronotal shield is present and represented by two weakly sclerotized shields situated around the bases of setae *d1*. Pygidial shield is apunctate, with the anterior margin indiscernible. Setae *d2*, *d1* and *e2* are long and subequal in length. Setae *h1* is distinctly longer (1.4 times) than *f1*. Length ratio of setae *f1*:*f2:* 1:1.8–2. Setae *f2* and *h2* are long and subequal in length. Genital setae *g1* and *g2* are subequal in length and 1.8 times longer than pseudanal setae *ps1* and *ps2*. Setae *ag2* is 1.9–2.6 times longer than setae *g1*. The length ratio of aggenital setae *ag1*:*ag2*:*ag3* is 1–1.6:1:1–1.6. Coxal fields are I–IV punctate, and coxal III–IV has sparse granulation on the anterior margin. Setae *3b* is 1.2–1.6 times shorter than *3c*, and the length ratio of setae *3b*:*l’RIII*:*3c* is 1.8–2.1:1:2.4–3. *Legs*. Fan-like setae *p*′ and *p*″ of legs III and IV with 10–11 tines. Setae *tc*″ of legs III and IV are subequal or distinctly (1.2 times) longer than setae *tc*′ of legs III–IV. *Lengths of setae*: *vi 125 (115–135), ve 180 (150–190), si* 285 (280–310), *se* 300 (300–310), *c1* (290–310), *c2* 300 (290–340), *d2* 330 (290–330), *d1* 335 (310–350), *e2* 330 (320–340), *f1* 190 (195–230), *f2* (370–400), *h1* (270–310), *h2* 450 (400–420), *ps1* 35 (35–45), *ps2* 45 (35–45), *g1* 90 (80–125), *g2* 80 (110–140), *ag1* 270 (260–275), *ag2* 240 (190), *ag3* 280 (250–290), *l*′*RIII* 45 (35–55), *l*′*RIV* 45 (45–50), *tc*′ *III–IV,* (90–100), *tc*″ (105–110), *3b* (90–105), and *3c* (135–150). 

Male (paratype, Figure 5 and Figure 6). Total body length is 835. *Gnathosoma*. Infracapitulum apunctate. Each medial branch has three chambers, each lateral branch has 10 chambers. The lengths of stylophore and movable cheliceral digits are 195 and 185, respectively. The propodonotal shield is weakly sclerotized and apunctate, sculptured in the lateral and central part of this shield. Setae *se* is situated out of this shield, setae *c2* is posterior to level setae *si*. Near the bases of setae *vi*, *ve*, *si*, there is a visibly granulated surface. The length ratio of setae *vi*:*ve*:*si* is 1:1.2:4. The hysteronotal shield is not fused to the pygidial and is semicircular on the posterior margin, bearing the bases of setae *d1* and *e2*. The length ratio of setae *d2*:*d1*:*e2* is 1.5:1.2:1. Setae *f2* is 4.8 times shorter than setae *h2*. The pygidial shield is weakly sclerotized with an indistinct anterior margin. The coxal fields of setae I–IV are apunctate. Setae *3b* is 1.7 times shorter than setae *3c*. Lengths of setae: *vi,* 45, *ve* 55, *si* 180, *se* 175, *c1* 165, *c2* 165, *d2* 55, *d1* 45, *e2* 35, *f2* 40, *h2* 195, *ag1* 80, *ag2* 60, *ag3* 80, 3b 55, and 3c 95.

##### Host and Distribution

Birds of the family Alaudidae: the dune lark, *Calendulauda erythrochlamys* (Strickland) from Namibia.

##### Type Material

The types of material included a female holotype, with ten female and one male paratype from the quill of the dune lark *Calendulauda erythrochlamys* (Strickland) (Passeriformes: Passerellidae), NAMIBIA, Karas, Waaihoek, 26°16′27″ S, 16°26′27″ E, 1116m a.s.l., 27 August 2009, coll. Komen, (USNM 642442). The Glowska-Patyniak E. sampled mites, vouchers, and DNA codes are as follows: KR028 and KR029. DNA barcode GenBank accession number PQ677794.

##### Type Material Deposition

Female holotype (USNMENT acc. number: USNMENT01967028) and five paratypes (four females and one male) (USNMENT01967029–USNMENT01967033) are deposited in the USNM, and the four female paratypes are in the AMU (EG24-0927-001.01-05).

##### Differential Diagnosis

*S. erythrochlamys* sp. n. is morphologically similar to *Syringophilipsis empidonax* Skoracki, Flannery and Spicer, 2008 described from the Hammond’s flycatcher *Empidonax hammondii* (Vesey, 1858) (Passeriformes: Tyrannidae) from Texas, USA [33]. In both species, females have a hysteronotal shield consisting of two sclerites surrounding the bases of setae *d1*. The pygidial shield is apunctate, and setae *f1* and *h1* are unequal in length. This new species is distinguished from *S. empidonax* by the following characters: in females of *S. erythrochlamys* sp. n., the propodonotal setae *vi*, *ve*, *si* are long and measure 115–135, 150–190, and 280–310, respectively. Setae *h1* 1.4 times longer than setae *f1*. The coxal fields I–IV are sparsely punctate. Genital setae *g1* are 1.2–1.6 times shorter than setae *ag2*. In females of *S*. *empidonax*, the propodonotal setae *vi*, *ve*, *si* are relatively short and measure 55–70, 80–90,170–200, respectively. Setae *h1* is more than two times longer than setae *f1*. The coxal fields I–IV are apunctate. The genital setae *g1* are 4.1–5.5 times shorter than setae *ag2*.

##### Etymology

The name is taken from the specific name of the host and is a noun in apposition.

**Figure 7:**
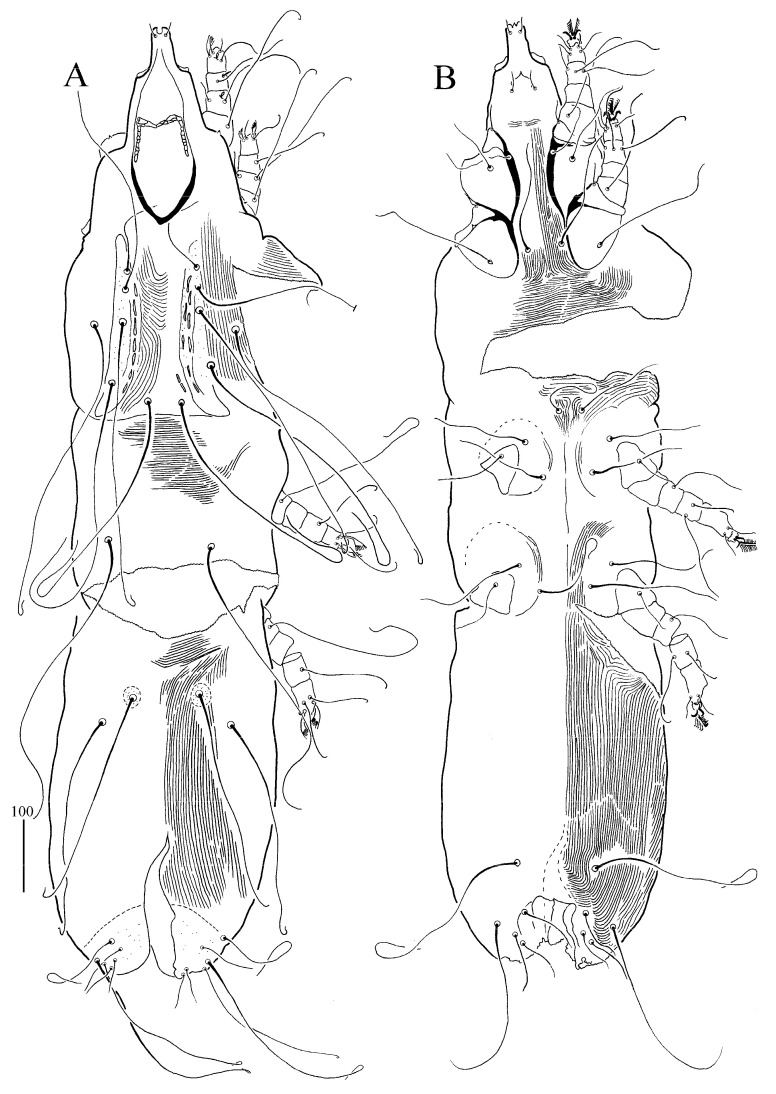
(**A**,**B**) *Syringophilopsis christinae* sp. n., female: (**A**) dorsal view, (**B**) ventral view.

**Figure 8:**
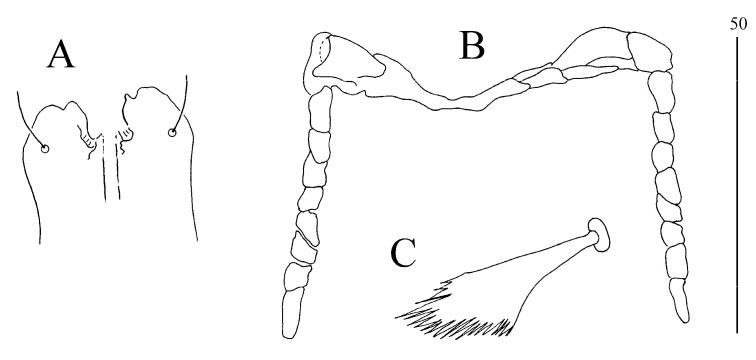
(**A**–**C**) *Syringophilopsis christinae* sp. n., female: (**A**) hypostomal apex, (**B**) peritremes, (**C**) fan-like setae p′ of leg III.

Syringophilopsis christinae sp. n. (Figure 7 and Figure 8)

Female (holotype and eight paratypes, range in parentheses). The total body length is 1330 (1185–1270). Gnathosoma. There is a hypostomal apex with two-minute protoburances. Infracapitulum apunctate. Each medial branch of the peritremes has three chambers, and each lateral branch has 8–9 chambers. The lengths of the stylophore and the movable cheliceral digit are 270 (230–270) and 190 (190–205), respectively. Idiosoma. The propodonotal shield is deeply concave on the anterior margin, with a visible scopulate on the lateral and central part, and punctate near the bases of setae *ve* and *si*. The bases of setae *si* and *c2* are situated at the same transverse level. The length ratio of setae *vi*:*ve*:*si* is 1:2.8–3:4–4.5. The hysteronotal shield is represented by two weakly sclerotized and densely punctate sclerites situated around the bases of setae *d1*. The pygidial shield is present and densely punctate. Setae *d2*, *d1,* and *e2* are long and subequal in length. Setae *h1* and *f1* are subequal in length and 1.6–2 times shorter than *f2* and *h2*. The genital setae *g1* and *g2* are subequal in length, and both are 1.4–1.8 times shorter than *ag2* and 1.2–1.8 times longer than the pseudanal setae *ps1* and *ps2*. Setae *ag1* and *ag3* are similar in length, and both are 1.8–2.3 times longer than *ag2*. The coxal fields I–IV are sparsely punctate, and setae *3b* and *3c* are subequal in length and 2.1–2.9 times longer than *l*′*RIII*. *Legs*. There are an-like seta *p*′ and *p*″ of legs III–IV, with 14–15 tines. Setae *tc*′ and *tc*″ of legs III and IV are subequal in length. Lengths of setae: *vi* 110 (105–115), *ve* 310 (315–370), *si* 455 (440–460), *se* 485 (420–490), *c1* (440–480), *c2* 490 (430–490), *d2* 525 (455–480), *d1* 570 (480–550), *e2* 545 (490–515), *f1* 250 (205–250), *f2* (440–490), *h1* 250 (230–285), *h2* (470–510), *ps1* (45–55), *ps2* 55 (40–60), *g1* 95 (70–100), *g2* 80 (70–90), *ag1* 290 (270–275), *ag2* 110 (130–160), *ag3* (250–310), *l*′*RIII* 55 (50–60), *l*′*RIV* 50 (45–60), *tc*′ III–IV 70 (75–90), *tc*″ III–IV 85 (70–90), 3b (120–135), and 3c (130–160).

Male: not found.

##### Host and Distribution

Birds of the family Alaudidae: the Karoo long-billed lark *Certhilauda subcoronata* (Smith) and the spike-heeled lark *Chersomanes albofasciata* (de Lafresnaye) from Namibia.

##### Type Material

Female holotype and eight female paratypes from the quill of the Laroo long-billed lark *Certhilauda subcoronata* (Smith) (Passeriformes: Alaudidae), NAMIBIA, Karas Aus Townlands, 26°37′32″ S, 16°16′35″ E, 1400m a.s.l., 24 August 2009, coll. Schmidt B.K. (USNM 642424). Mites were sampled by Glowska-Patyniak E. (September 2013), and the vouchers and DNA codes are as follows: KR025-KR027.

##### Additional Material

Eight females from the spike-heeled lark *Chersomanes albofasciata* (de Lafresnaye) (Passeriformes: Alaudidae), NAMIBIA, Karas, Kolka, 27°35′51″ S, 16°52′46″ E, 1300m a.s.l., 22 August 2009, coll. Komen, J. (USNM 642561). Mites were sampled by Glowska-Patyniak E. (September 2013), and the vouchers and DNA codes are as follows: KR067-KR069.

##### Type and Additional Material Deposition

Female holotype (USNMENT acc. number: USNMENT01967034), four paratypes (USNMENT01967035-USNMENT01967038), and four females (USNMENT01967039-USNMENT01967042) of additional material are deposited in the USNM. Four female paratypes (EG24-0927-002.01-04) and four females of add. mat. (EG24-0927-003.01-04) are in the AMU.

##### Differential Diagnosis

*Syringophilopsis christinae* sp. n. is the most morphologically similar to *Syringophilopsis fringillae* (Fritsch, 1958), described from *Fringilla coelebs* (Passeriformes: Fringillidae) from Germany. In both species, females have a hysteronotal shield represented by two sclerites surrounding the bases of setae *d1*. The pygidial shield is sparsely punctated, and setae *f1* and *h1* are shorter than setae *f2* and *h2*. The bases of setae *se* are situated distinctly anterior to the level setae *c2*. The coxal fields III–IV are sparsely punctate. This new species is distinguished from *S. fringillae* by the following characters: in females of *S. christinae* sp. n., the lateral branches of the peritremes have 8–9 chambers, and the length ratio of the propodonotal setae *vi*:*ve*:*si* is 1:2.8–3:4–4.5. Setae *h1* and *f1* are subequal in length and 1.6–2 times shorter than *h2* and *f2*. The hysteronotal shield is sparsely punctate. Setae *3c* is subequal or distinctly longer (1.3 times) than setae *3b*. Setae *ag2* is 1.4–1.8 times longer than the genital setae *g1* and 1.8–2.3 times shorter than setae *ag1* and *ag3*. In females of *S. fringillae,* the lateral branches of the peritremes have 13–14 chambers, and the length ratio of the propodonotal setae *vi*:*ve*:*si* is 1:1.5:1.5–2. Setae *h1* is 1.8–2 times longer than *f1*, and both are 1.3–1.4 times shorter than *h2* and *f2*. The hysteronotal shield is apunctate. Setae *3c* is 1.5 times longer than setae *3b*. Setae *ag2* is 2.8–3 times longer than the genital setae *g1* and 1.2 times shorter than setae *ag1* and *ag3*.

##### Etymology

This species is named in honor of the American ornithologist Christina Gebhard.

#### 3.2.3. Other Species

##### *Syringophilopsis acrocephali* Skoracki, 1999

*Syringophilopsis acrocephali* Skoracki, 1999: 160, Figures 8–10.
Type host: *Acrocephalus scirpaceus* (Hermann) (Passeriformes: Acrocephalidae). Type locality: Poland.

##### Host and Distribution

Birds of the family Acrocephalidae: the Eurasian reed warbler *Acrocephalus scirpaceus* (Hermann) from Poland and Russia [31,34], the marsh warbler *A. palustris* (Bechstein), and the sedge warbler *A. schoenobaenus* (Linnaeus), both of which are from Poland and Slovakia [31].

##### Material Examined

Five females from the quill of the African reed warbler *Acrocephalus baeticatus* (Vieillot, LJP) (Passeriformes: Acrocephalidae), NAMIBIA, Karas, Sandfontein near the Orange River, 28°51′45” S 18°33′08” E, 20 August 2009, coll. Gebhard, USNM 642547. Mites were sampled by Glowska-Patyniak E. (September 2013). Specimen vouchers and DNA codes: EP220, EP256, EP257 and KR064. DNA barcode GenBank accession number: PQ677793.

##### Material Deposition

Three females (USNMENT01967043–USNMENT01967045) are deposited in the USNM and two females are in the AMU (EG24-0927-004.01-02).

## 4. Discussion

This work proposes a new approach to researching quill mites’ diversity, ecology, and importance in epidemics. It involves comprehensive morphological and molecular systematics complemented by data on microbial associations. Mites are partners in a three-level system (bird host—quill mites—bacteria), each affecting the other. While the external morphology is generally effective in systematic studies, using molecular biology tools for analysis enhances our understanding of this complex system. Out of nearly five hundred species of quill mites described so far [7], only a small fraction have undergone parallel morphological and molecular analyses [35,36,37]. Even fewer species have been studied for their microbiological significance [4,5]. The main reason for this is that most research on these parasites has relied on museum specimens, which are often inadequately preserved for molecular analysis. As a result, such materials are considered unsuitable for DNA studies. Our results contradict this assumption and indicate that valuable results can be obtained even from several-year-old museum specimens that have been dried and stored at room temperature. The efficiency of using such materials is generally lower than analyzing “fresh” specimens, primarily due to the partial degradation of the genetic material. However, they can still yield reliable morphological and molecular information. This is a positive development for the future, especially for unique museum specimens that are difficult or impossible to obtain from nature.

Namibia is home to approximately seven hundred species of birds, many of which are endemic [32]. While there have been several reports of syringophilids from this region, with some species described [37,38], these findings only highlight the significant gap in our understanding of the diversity of these parasites in the area. Additionally, quill mites found on African birds have yet to undergo microbiological analysis. Previous studies indicate that mites parasitizing birds in Poland are hosts to unique phylogenetic lineages of endosymbiotic bacteria belonging to the genera *Wolbachia* and *Spiroplasma* [5]. Further study of this system, especially considering the parasites of endemic birds from different zoogeographic regions, may enhance our understanding of endosymbiont diversity.

Here, we conducted a comprehensive microbiological study on quill mites that parasitize Namibian birds, specifically three species of alaudids found only in southern Africa, as well as an acrocephalid representative widely distributed across Africa and the Arabian Peninsula. We detected *Wolbachia* only in a few individuals of *Syringophilopsis christinae* sp. n., collected from the Karoo long-billed lark and the spike-heeled lark. We identified one ASV annotated as *Wolbachia*, which is 100% identical to sequences previously isolated from two species of quill mites: *Torotrogla merulae* and *T. cardueli*. These strains were classified as supergroups P and Q based on analysis of five genes: 16S rRNA, *coxA*, *ftsZ*, *gltA*, and *groEL* [4]. Further investigation and sequencing of additional loci are necessary to accurately identify the supergroup infecting mites in Namibia. *Wolbachia* abundance was relatively lower than in other W+ samples previously reported from Poland [5]. This may be due to the low prevalence/abundance of the endosymbiont, but it may also result from the limited usability of the museum specimens. We found no ASVs of *Spiroplasma* or other well-known endosymbionts in Namibian mites.

We have previously speculated on the potential phenotypic effects that *Wolbachia* can induce in quill mites [4,5]. The most likely effect appears to be the manipulation of reproductive functions, resulting in a significantly unbalanced sex ratio. This imbalance is characterized by a predominance of females and, in extreme cases, a complete absence of males in the population. Quill mites obtain food by suctioning tissue fluids. Thus, it is also possible that these endosymbionts act as digestive aids by providing essential nutrients [39,40]. Understanding the impact of endosymbionts on the biology of *Syringophilopsis christinae* sp. n. is challenging. Since *Wolbachia* is not found in all individuals and, where it does occur, is present with low abundance, it does not seem to be an essential food endosymbiont. Manipulating the sex ratio seems possible, as only one male was found in all the analyzed species or populations, where numerous females were observed. However, *Wolbachia* was found only in the *S. christinae* sp. n. population. At this point, drawing definitive conclusions about the endosymbiont’s role in this mite species remains difficult, indicating the need for more research on this system.

The quill mites were also examined for their significance regarding epidemic potential. Due to the way that quill mites take in food (pierce the quill with chelicerae and suck out the bird’s tissues), mites, similarly to ticks, can pose a threat to birds. Previous studies have suggested that mites may carry potential pathogens from the genera *Anaplasma*, *Brucella*, and *Bartonella* [5,15]. Our analysis did not find any ASVs representing these known pathogenic bacteria. Further research is necessary to consider a broader diversity of species and populations.

The other bacterial taxa revealed in our studies were previously found in quill mites [5], but the impact on their biology remains unclear. Some bacteria may be environmentally acquired (derived from habitat or food). The mites inhabit the quill, interacting with the host’s microbiome, including the skin, feathers, and tissue fluids. The composition of the mite microbiome may, therefore, partly reflect their environment. We found bacterial taxa that were previously noted in the skin and feather microbiome of birds, e.g., Corynebacteriaceae, Enterobacteriaceae, Methylobacteriaceae, Pseudomonadaceae, Sphingomonadaceae, Staphylococcaceae, Streptococcaceae, and Xanthomonadaceae [41,42,43]. This may suggest the sharing of microbiomes between the bird host and parasites.

Our study’s findings include two new-to-science species described here. *Syringophilopsis* Kethley, 1970 is among the most numerous and widespread genera of quill mites, with over fifty species having been discovered worldwide [31,33,44]. In Africa, twelve species of *Syringophilopsis* have been described from birds belonging to two orders, Passeriformes and Coraciiformes [45,46]. None of these species have been recorded in Namibia. Knowledge of the diversity of quill mites in this country is nearly non-existent. Currently, only four species of mites that parasitize four bird species have been documented in this area [38,47]. Therefore, perspectives from conducting a comprehensive parasitological analysis of all seven hundred host species occurring there seem very distant at this time. Only one species, *Torotrogla paenae* Glowska, Romanowska, Schmidt et Dabert, 2018, parasitizing the Kalahari scrub robin *Cercotrichas paena* (Passeriformes: Muscicapidae) was described in Namibia based on morphological and molecular data [37]. The value of DNA barcoding has been demonstrated multiple times in many groups of arthropods [48,49], including quill mites [36,50]. It has uncovered phenomena such as phenotypic plasticity, dimorphic females, and cryptic species [12,13,14]. Here, we subjected all available material to DNA barcoding analysis, however, its efficiency was quite low. Out of the twenty-one analyzed specimens representing three mite species, we obtained only six high-quality COI sequences, which have been assigned to two species, i.e., *S. erythrochlamys* sp. n. and *S. acrocephali*. This may be attributed to DNA degradation in the poorly preserved museum specimens.

In this study, we proposed a novel approach to systematically investigate mites by incorporating morphometry, DNA barcoding, and microbiome studies. By adding a microbiological dimension, we can evaluate the significance of mites as hosts for endosymbionts and explore their role in the transmission of potential pathogens among birds. Our findings enhance our understanding of the species richness of quill mites and their relationships with hosts in Namibia, an area that has been extremely poorly understood. In future research, we encourage a multidimensional systematic and microbiological analysis of quill mites.

## 5. Conclusions

Knowledge of quill mites is increasing, yet only about 10% of their global diversity has been described so far. The parasitofauna of birds in African countries, including Namibia, remains one of the least understood. Recently, there has been a call for systematic studies that incorporate classical morphological approaches and consider molecular data. DNA barcoding helps estimate genetic diversity and investigate host specificity. It also reveals issues related to misinterpretations of morphological features, including polymorphisms and cryptic species. Accurate species identification is crucial for advancing research, particularly to understand parasites’ ecological relationships and epidemiological significance. Recent reports indicate that quill mites host unique phylogenetic lineages of endosymbiotic bacteria, including *Wolbachia* and *Spiroplasma*. These mites are also considered vectors that can potentially transmit pathogens between birds. To address this, we proposed a new, comprehensive approach to analysing quill mite material, allowing for simultaneous examination of various aspects including morphological and DNA-based systematics, as well as complementary data on microbial associations. Our research led to the discovery of two new species of mites that parasitize birds in Namibia, providing new insights into *Wolbachia* infections among quill mites in Africa. Additionally, microbiome analysis revealed no bacterial taxa that are commonly deemed harmful to birds. Overall, our research enhances the understanding of mite diversity and lays a foundation for future ecological and epidemiological studies.

## Figures and Tables

**Table 1:** Overview of quill mites sampled for the study with a relative abundance of *Wolbachia*. The first value refers to the relative abundance of all five mites in the microbial communities taken together, and the second value (in brackets) refers to the same measure in the microbial communities of the two Wolbachia-infected mites.

Quill Mite Species	Bird Host Species (Common Name)	Bird Host Order and Family	No. of Mite Individuals (W+)	Relative *Wolbachia* Abundance %
*Syringophilopsis christinae* sp. n	Karoo long-billed lark *Certhilauda subcoronata* Smith	Passeriformes: Alaudidae	5 (2)	0.11% (0.33%)
	Spike-heeled lark *Chersomanes albofasciata* (de Lafresnaye, NFAA)		5 (2)	0.25% (0.67%)
*Syringophilopsis erythrochlamys* sp. n.	Dune lark *Calendulauda erythrochlamys* (Strickland, HE)	Passeriformes: Alaudidae	5 (0)	0%
*S. acrocephali* Skoracki, 1999	African reed warbler *Acrocephalus baeticatus* (Vieillot, LJP) (new host)	Passeriformes: Acrocephalidae	6 (0)	0%

## Data Availability

Data are available upon request from the corresponding author.
